# Global research progress on obstructive sleep apnea in the field of radiological imaging: bibliometric and visualization analysis

**DOI:** 10.3389/fneur.2025.1494359

**Published:** 2025-06-13

**Authors:** Yi Zhang, Xiaoyuan Chai, Peng Sun, Xiangsheng Li

**Affiliations:** ^1^Postgraduate Training Base of Air Force Medical Center, China Medical University, Beijing, China; ^2^Department of Radiology, Air Force Medical Center, Air Force Medical University, Beijing, China

**Keywords:** obstructive sleep apnea-hypopnea syndrome, imaging, CiteSpace, bibliometric analysis, visual analysis

## Abstract

**Background:**

This study aims to assess the intersection of OSA with radiological imaging through bibliometric analysis, exploring research trends and emerging hotspots to identify future research directions.

**Methods:**

This study conducted a literature search for publications related to OSA and radiological imaging from 2004 to 2023 in the Web of Science database. We utilized tools such as CiteSpace, R language, and VOSviewer for bibliometric analysis to explore key trends and hotspots in this interdisciplinary field.

**Results:**

The search yielded 2,000 publications, showing a fluctuating upward trend in publication volume. Research predominantly originates from countries/regions such as the United States, China, and Turkey. Leading journals in this field include Sleep, the American Journal of Respiratory and Critical Care Medicine, and Chest; with 7,202 research institutions involved, notable ones include the University of California system, Harvard University, the University System of Ohio, Capital Medical University, and Seoul National University. A total of 10,099 researchers have contributed to this field, including prominent figures such as Kumar R, Harper RM, and Schwab RJ. Keyword analysis indicates current research hotspots are imaging diagnostic techniques for OSAHS, comorbidities associated with OSAHS, and the pathogenesis of OSAHS-related comorbidities.

**Conclusion:**

Currently, imaging studies on OSAHS are increasingly becoming a focal area, and new imaging diagnostic techniques for OSAHS require further validation through studies with larger sample sizes and more standardized methods. Additionally, research into the interactions between OSAHS and its comorbidities, different pathogenic mechanisms, and the integration of neuroimaging biomarkers with negative cognitive outcomes urgently needs to be deepened.

## 1 Introduction

Obstructive Sleep Apnea-Hypopnea Syndrome (OSAHS) is a widely common yet frequently underdiagnosed sleep disorder globally. According to a recent study conducted by the American Academy of Sleep Medicine, which employed the diagnostic criteria from 2012, an estimated 936 million adults, aged 30 to 69, suffer from OSA ranging from mild to severe. Within this demographic, 425 million adults are grappling with moderate to severe manifestations of this disorder (1). The main symptoms include nighttime snoring, apnea, frequent awakenings, and excessive daytime sleepiness. This condition not only severely affects the patient’s quality of life, but is also strongly associated with various health problems such as hypertension, cardiovascular disease, diabetes, and cognitive impairment (2, 3).

The pathophysiology of OSAHS is complex and multifactorial, resulting from the interaction between anatomical and non-anatomical factors. Current research has found that the primary pathogenic factor of OSAHS is the weakening of neuromuscular drive during sleep, which subsequently leads to reduced tension in the extrinsic tongue and pharyngeal dilator muscles, causing upper airway collapse. This is specifically manifested as the tongue falling back and downward, pharyngeal constriction, and airway obstruction. The likelihood of this condition escalates if the patient exhibits a narrowed upper airway, a consequence of either excessive surrounding soft tissue or a diminutive craniofacial skeleton that limits available space (4).

Currently, polysomnography is regarded as the gold standard for diagnosing OSAHS (1), where the Apnea-Hypopnea Index (AHI) and the lowest oxygen saturation levels are key indicators for assessing the severity of the condition. However, the role of these indicators in indicating upper airway collapse is not significant. Imaging is pivotal in elucidating the etiology of upper airway constriction in OSAHS patients. Substantial evidence underscores the correlation between airway constriction observed in awake patients and the ensuing onset of airway collapse and apnea during sleep. In the process of imaging the nervous system, craniofacial structures, sinuses, and head and neck, radiologists’ discernment of distinctive morphological traits of the upper airway’s soft tissue and bone, along with airway constriction, may hint at an undetected diagnosis of OSAHS. Imaging using CT or MRI provides a comprehensive assessment of the etiology of OSAHS, supplementing clinical examinations, particularly in patients who have failed medical therapy and are being evaluated for surgery. Therefore, imaging plays an important role in screening for OSAHS, clarifying the etiology, understanding the severity of the disease, selecting treatment options, and predicting prognosis.

Bibliometrics, as a quantitative analysis tool, can reveal the development trends, hotspots, and frontier dynamics of a specific research field through statistical analysis of related literature (5). By conducting a bibliometric analysis of literature related to OSAHS and radiological imaging, we can gain a more comprehensive understanding of the current research status and development trends in this interdisciplinary field, thereby providing valuable references for future research. This study aims to systematically analyze academic literature related to OSAHS and radiological imaging over the past 20 years using bibliometric methods. The main research content includes annual publication trends, major research institutions and countries, core journals, hot topics, and their evolution. Through these analyses, we hope to reveal the current research status and future trend directions, providing useful references for related researchers.

## 2 Materials and methods

### 2.1 Literature resources

**Figure 1:**
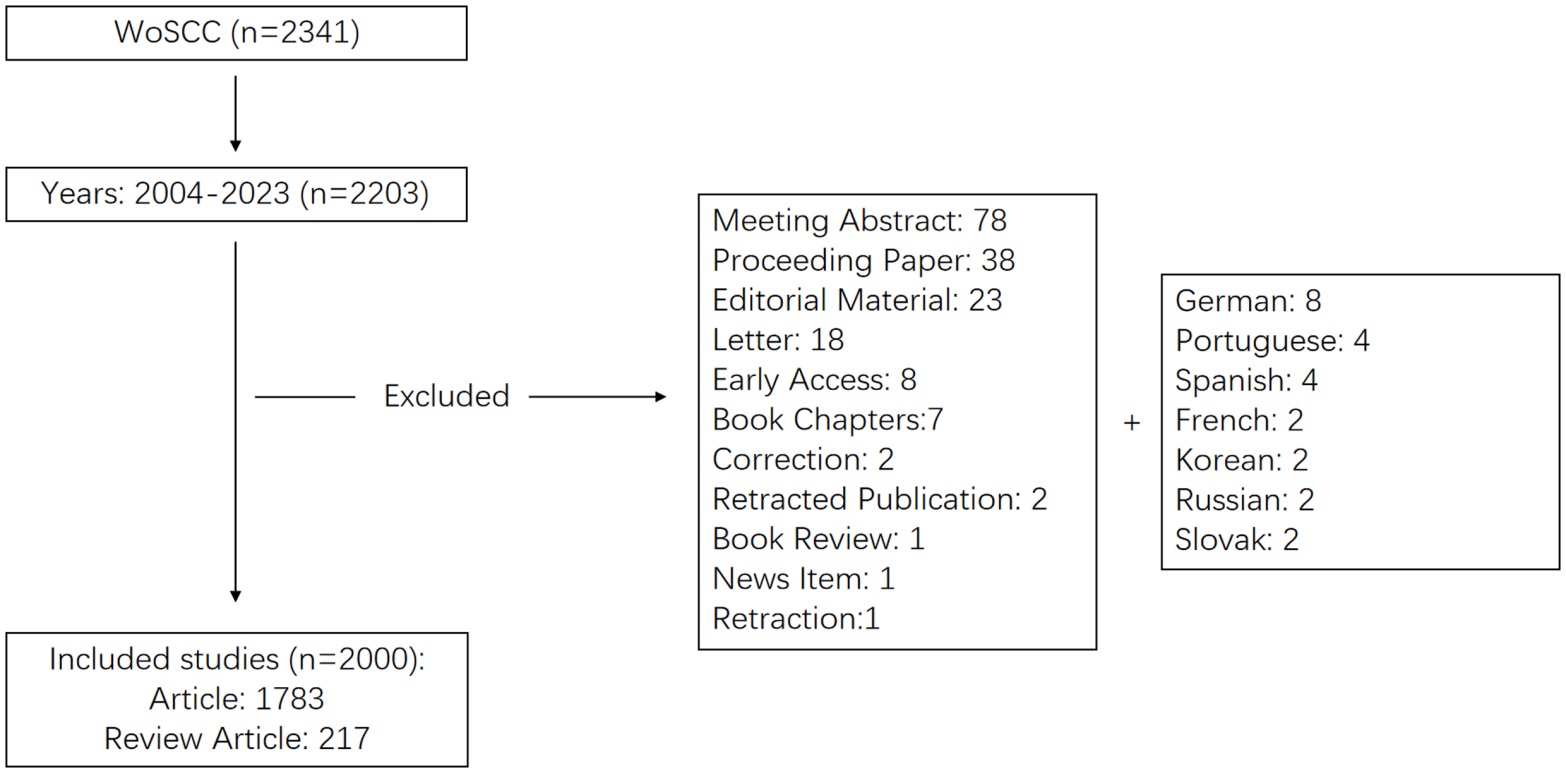
Flow diagram of the included articles.

A comprehensive literature search was conducted on the Web of Science Core Collection (WoSCC), a multidisciplinary database with an extensive citation network, for data pertinent to our field of study. The search strategy, as described in Supplementary material, combined subject terms and free-text keywords related to obstructive sleep apnea and medical imaging. The literature surveyed spanned from 2004 to 2023 and encompassed both original and review articles. The search terms included TS = (“obstructive sleep apnea,” “obstructive sleep apnea syndrome,” “obstructive sleep disorder,” “sleep apnea disorder,” “obesity hypoventilation syndrome”) and TS = (“magnetic resonance imaging” or “computed tomography” or “ultrasound imaging” or “imaging” or “tomography” or “medical imaging” or “medical imaging” or “radiology”). The final step entailed the exportation and storage of all retrieved documents in text format, thus facilitating subsequent bibliometric analyses. On May 15, 2024, two researchers independently executed the literature data search. The comprehensive search procedure is illustrated in Figure 1.

### 2.2 Literature analysis

The data analysis and management were conducted using CiteSpace 6.1. R2, VOS viewer 1.6.17, and Microsoft Office Excel 2010. Microsoft Office Excel 2010 software is capable of data management, annual publication tallying, and the creation of related tables. CiteSpace 6.1. R2 creates a visual map of annual publications, showing data on publication count, country, institution, author, keywords, and highly cited articles. VOS viewer 1.6.17, on the other hand, provides a graphical depiction of highly co-cited literature along with the co-occurrence of authors. CiteSpace settings and results match those of previous versions (6), where nodes can represent countries and institutions.

## 3 Results

### 3.1 Analysis of annual publications and publication trends

**Figure 2:**
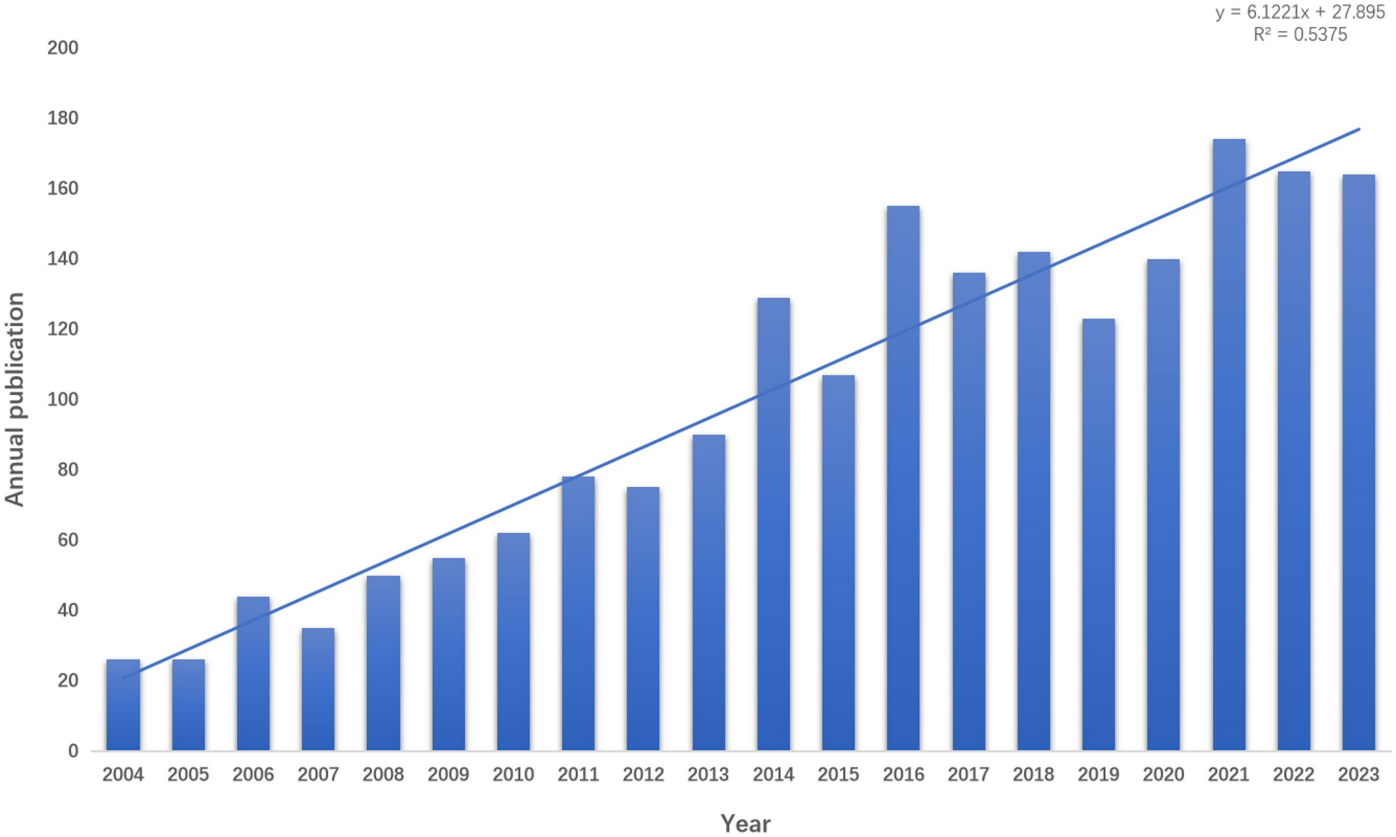
Trend chart of published research on osA and radiological imaging.

As of 31 December 2023, a total of 2,000 articles related to Obstructive Sleep apnea syndrome have been published in the field of radiological imaging, comprising 1,783 research articles and 217 review articles. The number of annual publications can be employed as a means of gauging trends in a specific research area. The analysis demonstrates a year-on-year increase in the number of papers published in this field, rising from 26 articles in 2004 to a peak of 174 articles in 2020 and 2021 (Figure 2). This indicates an escalating interest from researchers in the field. Moreover, the growth trend depicted in Figure 2, with a coefficient of determination (R^2^) of 0.5375, exhibits a positive correlation between the publication year and the count of publications. This suggests a continuing upward trajectory in the annual publication volume within this field.

### 3.2 Analysis of trends in countries, institutions, and authors

**Figure 3:**
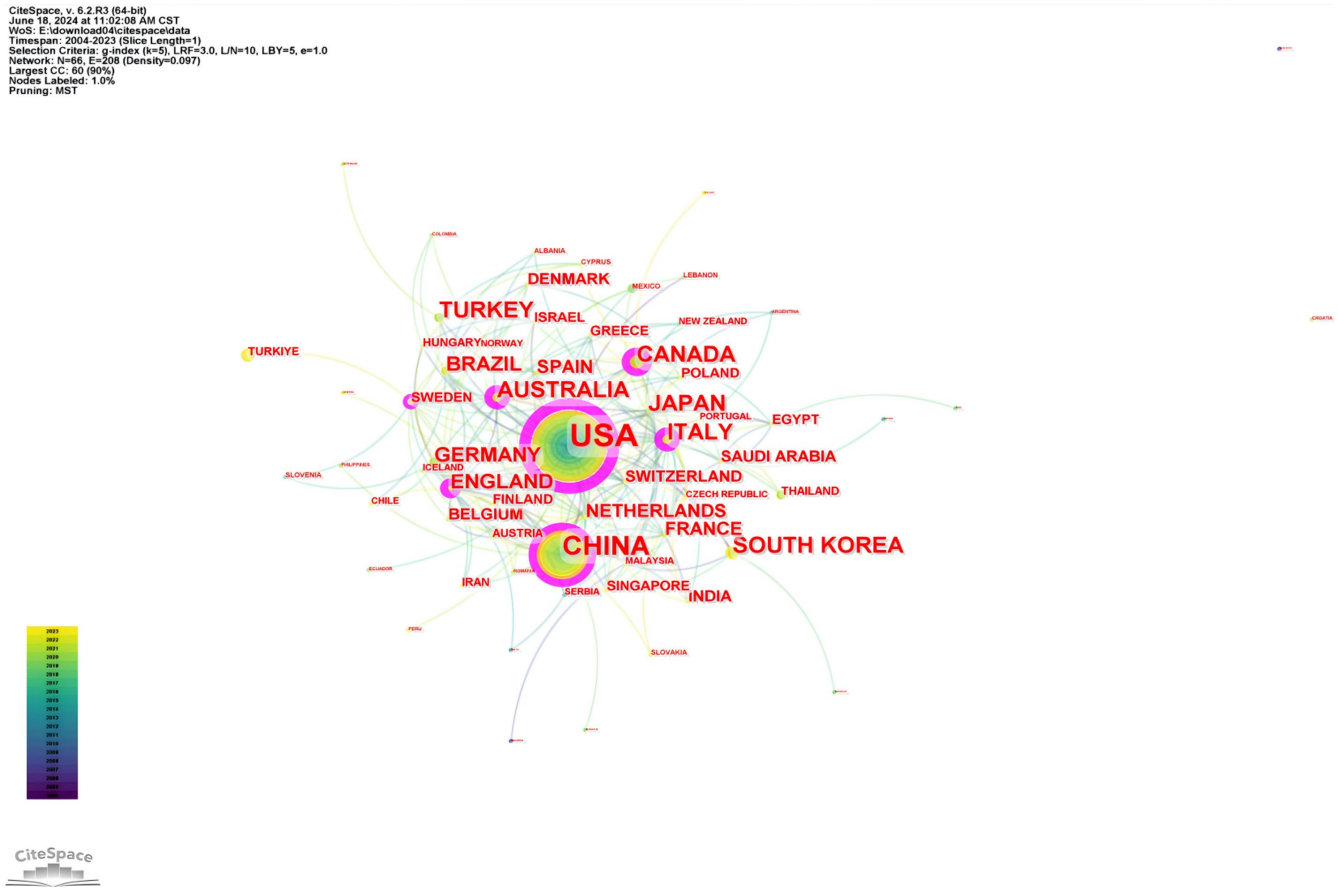
Country/region collaboration network in OSA and radiological imaging research. Created using CiteSpace.

The articles were disseminated in 376 countries and regions worldwide. The 74 nodes and 369 links represented in Figure 3 illustrate the geographical distribution of countries and the nature of international collaboration. In the graph, the node size reflects the volume of publications a country has contributed to a specified research area. If the centrality surpasses 0.1, a purple circle will augment the corresponding node on the network map. Table 1 enumerates the top 10 countries based on their publication count and centrality. The United States leads in terms of the number of published papers, with 704 publications accounting for 35.2% of the total, followed by China with 382 publications (19.1%), and Turkey with 133 publications (6.65%). These countries are all at the forefront of research in obstructive sleep apnea syndrome and imaging. A positive correlation exists between the degree of international collaboration and centrality. The findings reveal that the United States (0.48), China (0.15), Turkey (0.14), Australia (0.14), and Japan (0.12) rank as the top five countries in terms of centrality.

**Table 1:** Countries/regions, institutions, and authors ranked by publications and centrality.

Item	Rank	Name	Publications	Name
Countries/Regions	1	USA	704	USA
	2	China	382	China
	3	Turkey	133	Canada
	4	Australia	119	Sweden
	5	Japan	119	Australia
	6	South Korea	116	England
	7	Italy	100	Italy
	8	Canada	94	Netherlands
	9	England	81	Brazil
	10	Brazil	78	Japan
Institutions	1	University of California System	97	Harvard University
	2	Harvard University	40	University of California System
	3	Capital Medical University	39	University System of Ohio
	4	University of Pennsylvania	38	Harvard Medical School
	5	University of California Los Angeles	38	Capital Medical University
	6	University System of Ohio	22	Seoul National University (SNU)
	7	Chang Gung Memorial Hospital	22	Stanford University
	8	Stanford University	21	Brigham and Women’s Hospital
	9	University of Sydney	21	University of California Los Angeles
	10	Harvard Medical School	17	University of Pennsylvania
Authors	1	Kumar R	38	Kumar R
	2	Harper RM	32	Harper RM
	3	Schwab RJ	26	Schwab RJ
	4	Macey PM	26	Macey PM
	5	Woo MA	24	Woo MA
	6	Keenan BT	18	Keenan BT
	7	Bilston LE	17	Bilston LE
	8	Cistulli PA	16	Cistulli PA
	9	Shin C	16	Shin C

**Figure 4:**
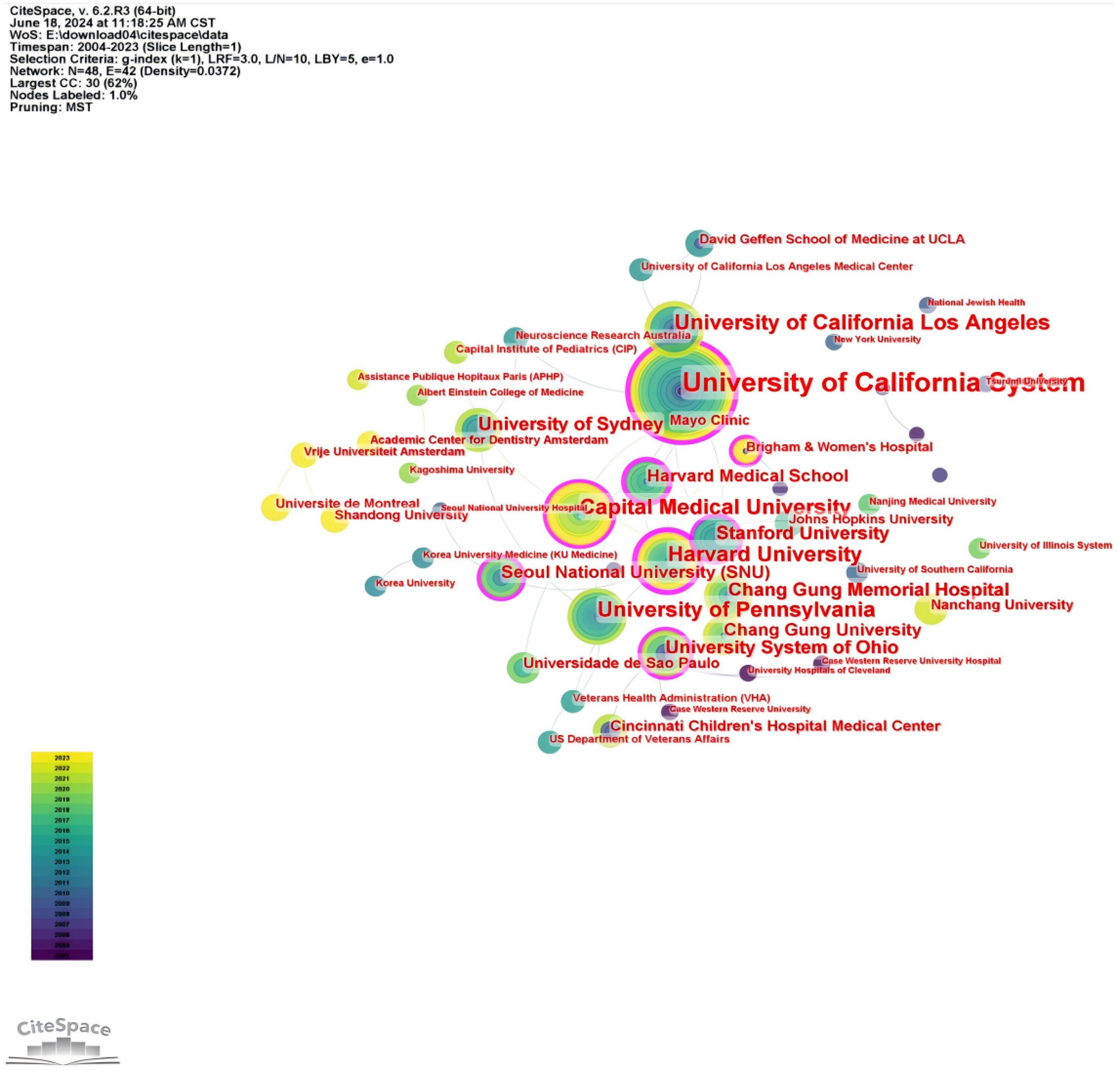
Research institution collaboration network related to OSA and radiological imaging. Created using Citespace.

A collective total of 7,202 institutions have contributed to this research field. Figure 4 delineates the collaborative networks among these institutions, embodying 473 nodes and 1,527 connections. Table 1 discloses that the top five universities in terms of publication count are the University of California System, with 97 publications (16.83%), and Harvard University, with 40 publications (13.86%). Capital Medical University (39 publications, 10.89%), University of Pennsylvania (38 publications, 9.90%), and University of California Los Angeles (38 publications, 8.91%). The five institutions exhibiting the highest centrality, indicative of the most extensive collaboration, encompass Harvard University (0.42), the University of California System (0.33), the University System of Ohio (0.20), Harvard Medical School (0.20), and Capital Medical University (0.16). These leading global universities and research institutions have significantly propelled progress in this field.

**Figure 5:**
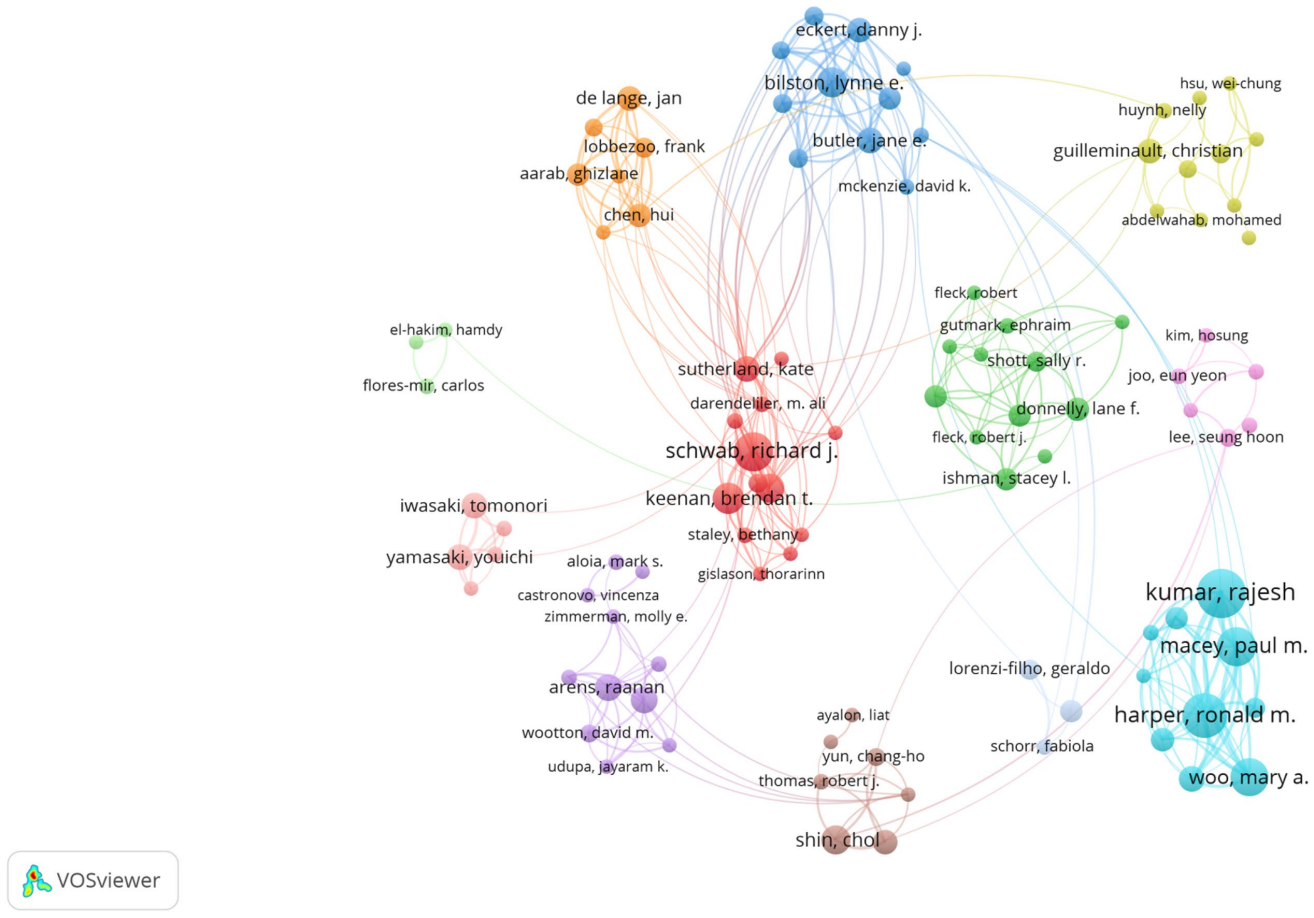
Author collaboration network related to OSA and radiological imaging research. Created using VoSviewer.

Figure 5 illustrates that 10,099 authors have published papers on the topic of obstructive sleep apnea and medical imaging. Table 1 enumerates the top five authors based on their respective counts of published articles. The four authors with the highest number of publications were Kumar R (38 publications, 8.91%), Harper RM (32 publications, 8.91%), Schwab RJ (26 publications, 8.91%), and Macey PM (26 publications, 8.91%). These were followed by Woo MA (24 publications, 15.79%). These five authors occupy significant positions within the field of obstructive sleep apnea and medical imaging research. The centrality of all authors is zero, suggesting a need for bolstering collaboration among authors.

### 3.3 Analysis of co-cited references

**Figure 6:**
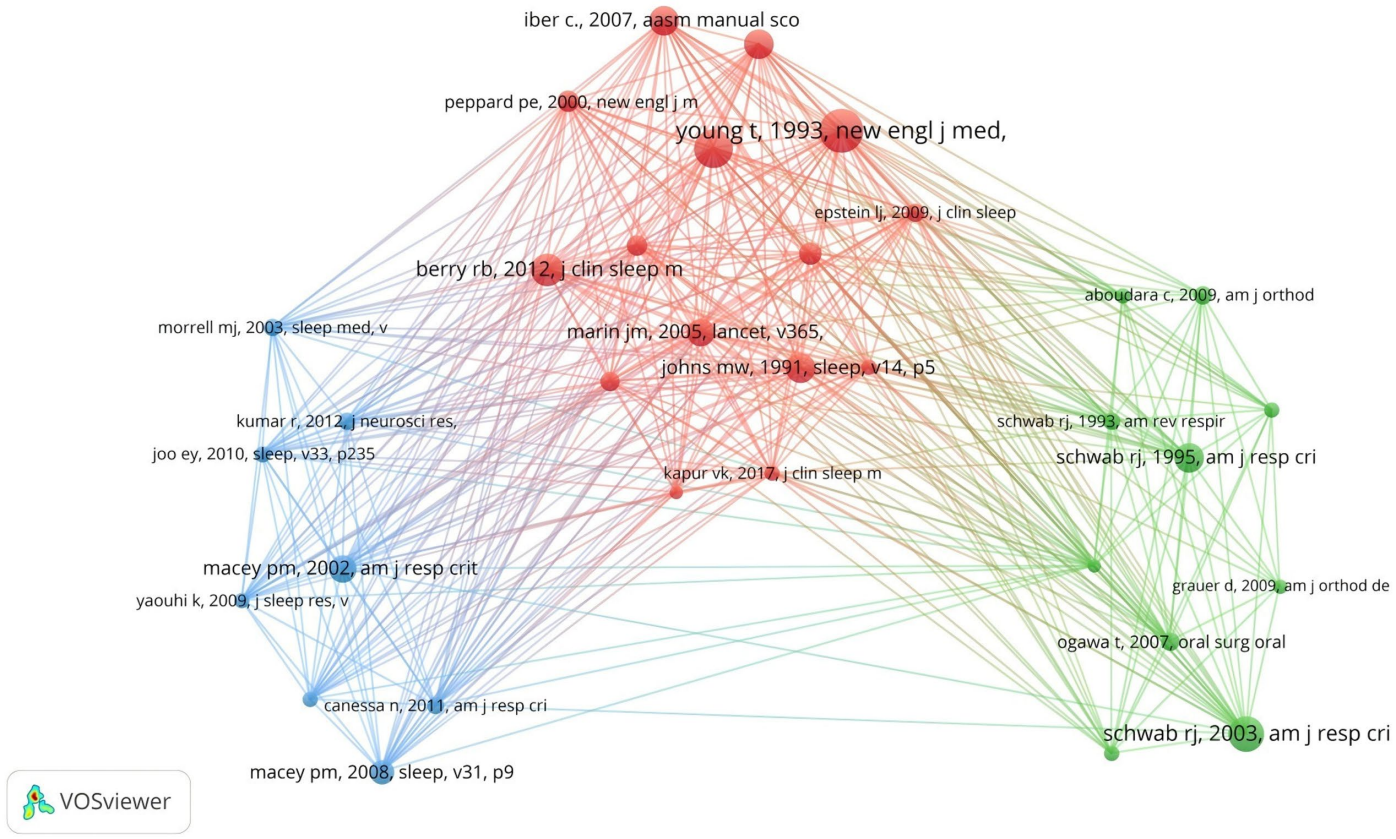
Visualization of a clustering map of co-cited references. Created with Vosviewer.

Co-cited references are those collectively cited by researchers. By analyzing these, VOSviewer visualizes the interconnections, emphasizing shared research areas between obstructive sleep apnea and medical imaging. As per VOSviewer’s analysis, a total of 53,446 references were cited in this field of study. When the citation threshold is lowered to 20, this number reduces to 291 references. Figure 6 reveals that these co-cited references are categorized into three clusters, each denoted by a distinct color on the visualization graph. The red cluster primarily highlights the assessment, diagnosis, measurement, and health impacts of OSA, including updates to the rules for evaluating sleep apnea events (7), clinical practice guidelines for diagnosing adult OSA (8), factors influencing daytime sleepiness (9), gender differences in the prevalence of sleep apnea (10), and the impact of severe OSA on cardiovascular events in men (11). The blue cluster mainly focuses on basic research evaluating the effects of OSA on brain regions and neural structures using medical imaging techniques (12–15). The green cluster’s literature primarily addresses the impact of OSA on the upper airway and soft tissue structures (16–19). Table 2 lists the top 10 most cited references, most of which come from the world’s leading journals in sleep medicine, such as the “New England Journal of Medicine” and “SLEEP.” Therefore, research on OSA in the field of radiological imaging is a current hotspot and frontier in sleep medicine.

**Table 2:** Top 10 highly co-cited references.

Item	Rank	Title	Citation	Year
Highly co-cited references	1	The occurrence of sleep-disordered breathing among middle-aged adults.	160	1993
	2	Sleep-related breathing disorders in adults: recommendations for syndrome definition and measurement techniques in clinical research.	141	1999
	3	Identification of upper airway anatomic risk factors for obstructive sleep apnea with volumetric magnetic resonance imaging.	131	2003
	4	Rules for scoring respiratory events in sleep: update of the 2007 AASM Manual for the Scoring of Sleep and Associated Events. Deliberations of the Sleep Apnea Definitions Task Force of the American Academy of Sleep Medicine	120	2012
	5	The visual scoring of sleep in adults.	108	
	6	A new method for measuring daytime sleepiness: the Epworth sleepiness scale.	108	1991
	7	Upper airway and soft tissue anatomy in normal subjects and patients with sleep-disordered breathing. Significance of the lateral pharyngeal walls.	108	1995
	8	Epidemiology of obstructive sleep apnea: a population health perspective.	106	2002
	9	Brain morphology associated with obstructive sleep apnea.	99	2002
	10	Long-term cardiovascular outcomes in men with obstructive sleep apnoea-hypopnoea with or without treatment with continuous positive airway pressure: an observational study.	99	2005

“Identification of upper airway anatomic risk factors for obstructive sleep apnea with volumetric magnetic resonance imaging” is the most cited paper published in the “American Journal of Respiratory and Critical Care Medicine” in the past 20 years (20). In this paper, Schwab et al. proposed that volumetric magnetic resonance imaging can be used to identify structural risk factors for obstructive sleep apnea. Furthermore, an examination of the top 10 most cited papers reveals that the majority originate from esteemed sleep medicine journals, such as the “New England Journal of Medicine” and “SLEEP.” This underscores the cutting-edge nature and innovation within this research field.

Furthermore, the cited literature over the past 20 years has focused on the impact of OSA on the anatomical structure of the upper airway and brain regions, as well as its health implications. Half of the top cited studies in the past 20 years are based on medical imaging analysis of the effects of OSA on the upper airway and brain regions. Schwab et al. found that volumetric magnetic resonance imaging can identify structural risk factors for obstructive sleep apnea, with the volume of the tongue and lateral pharyngeal walls being independent risk factors for OSA (19). Schwab et al.’s research also found that magnetic resonance imaging can visually show significant differences in upper airway size and surrounding soft tissue structures among apneic subjects, snorers, and normal subjects, but did not find a close relationship with parapharyngeal fat pads (19). Macey et al. observed that OSA leads to a reduction in gray matter volume in patients’ brains using high-resolution T1-weighted magnetic resonance imaging, with these gray matter loss areas occurring in regions involved in upper airway motor regulation and cognitive functions (13).

### 3.4 Analysis of keyword co-occurrence, clustering, and burst

**Figure 7:**
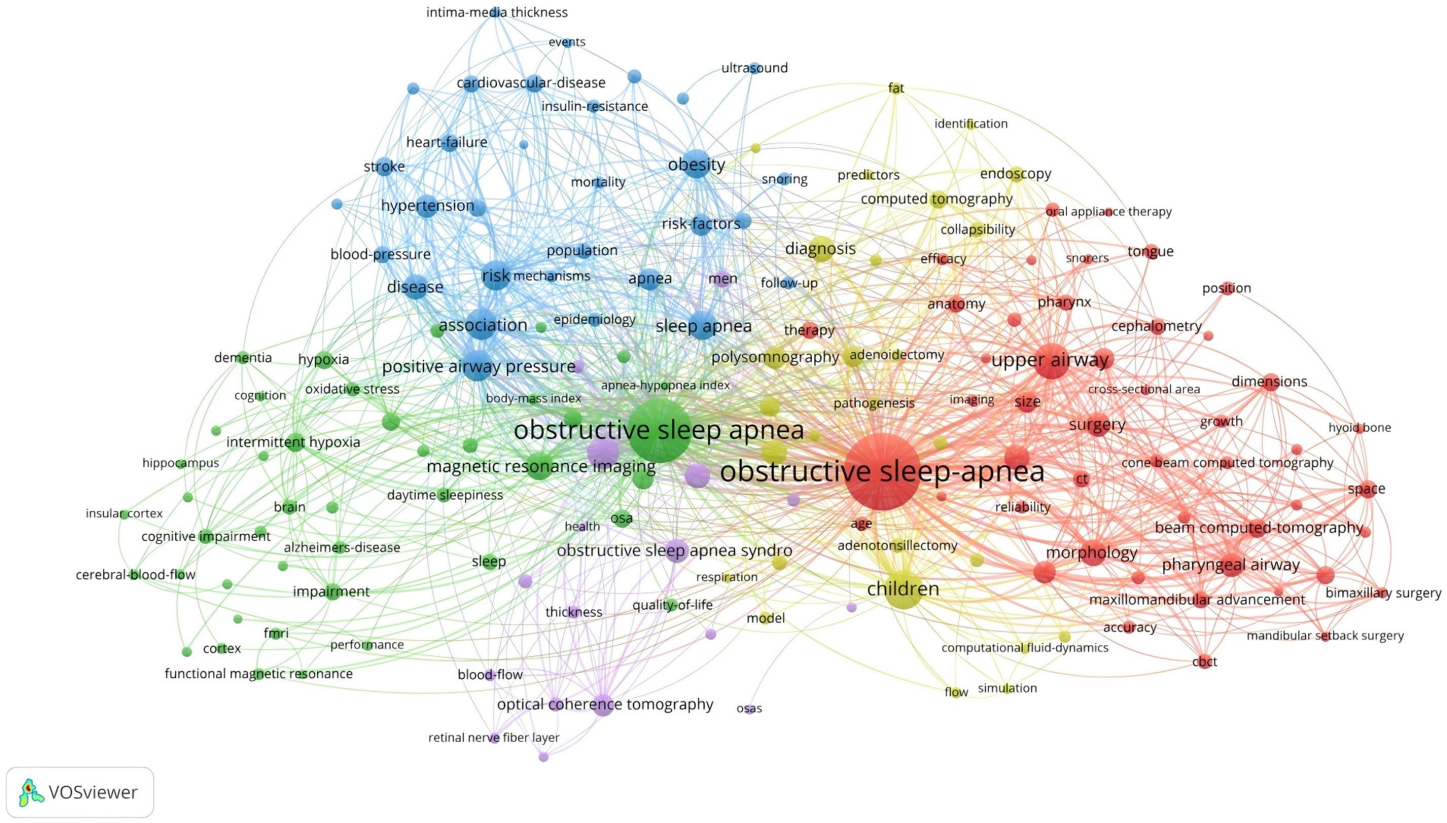
Keyword co-occurrence map of OSA and radiological imaging. Created with VOS viewer.

The co-occurrence of keywords provides insights into the topic and scope of the research field, as depicted in Figure 7. Table 3 displays the top 20 keywords based on their co-occurrence rate and centrality of obstructive sleep apnea and radiological imaging research from 2004 to 2023. “Obstructive sleep apnea” is the most frequently occurring keyword, followed by “upper airway” and “children.” More importantly, keywords such as “computed tomography,” “positive airway pressure,” “prevalence,” “magnetic resonance imaging,” “morphology,” “risk,” and “association” have been used more than 100 times, highlighting the current research hotspots and themes in this field. There is a positive correlation between centrality and the degree of linkage among keywords. In Table 3, “obstructive sleep apnea” is the most central keyword, followed by “computed tomography,” “magnetic resonance imaging,” “upper airway,” and “pharyngeal airway.” These keywords remain concentrated on the relationship between obstructive sleep apnea and the upper airway, as well as their connection with radiology.

**Table 3:** Top 10 highly co-cited references.

Rank	Keyword	Frequency	Keyword	Centrality
1	Obstructive sleep apnea	1,176	Obstructive sleep apnea	0.48
2	Upper airway	204	Upper airway	0.16
3	Children	188	Positive airway pressure	0.15
4	Sleep apnea	161	Magnetic resonance imaging	0.15
5	Computed tomography	160	Association	0.14
6	Positive airway pressure	151	Risk	0.12
7	Prevalence	150	Pharyngeal airway	0.12
8	Association	143	Children	0.11
9	Risk	137	Sleep apnea	0.11
10	Magnetic resonance imaging	120	Morphology	0.11
11	Morphology	103	Cardiovascular disease	0.11
12	Disease	99	Disease	0.1
13	Adults	98	Size	0.1
14	Pressure	86	Impairment	0.1
15	Pharyngeal airway	85	Computed tomography	0.09
16	Obstructive sleep apnea syndrome	85	Obstructive sleep apnea syndrome	0.09
17	Diagnosis	84	Adults	0.07
18	Obesity	78	Optical coherence tomography	0.07
19	Risk factors	74	CT	0.06
20	Surgery	70	Cognitive impairment	0.06

**Figure 8:**
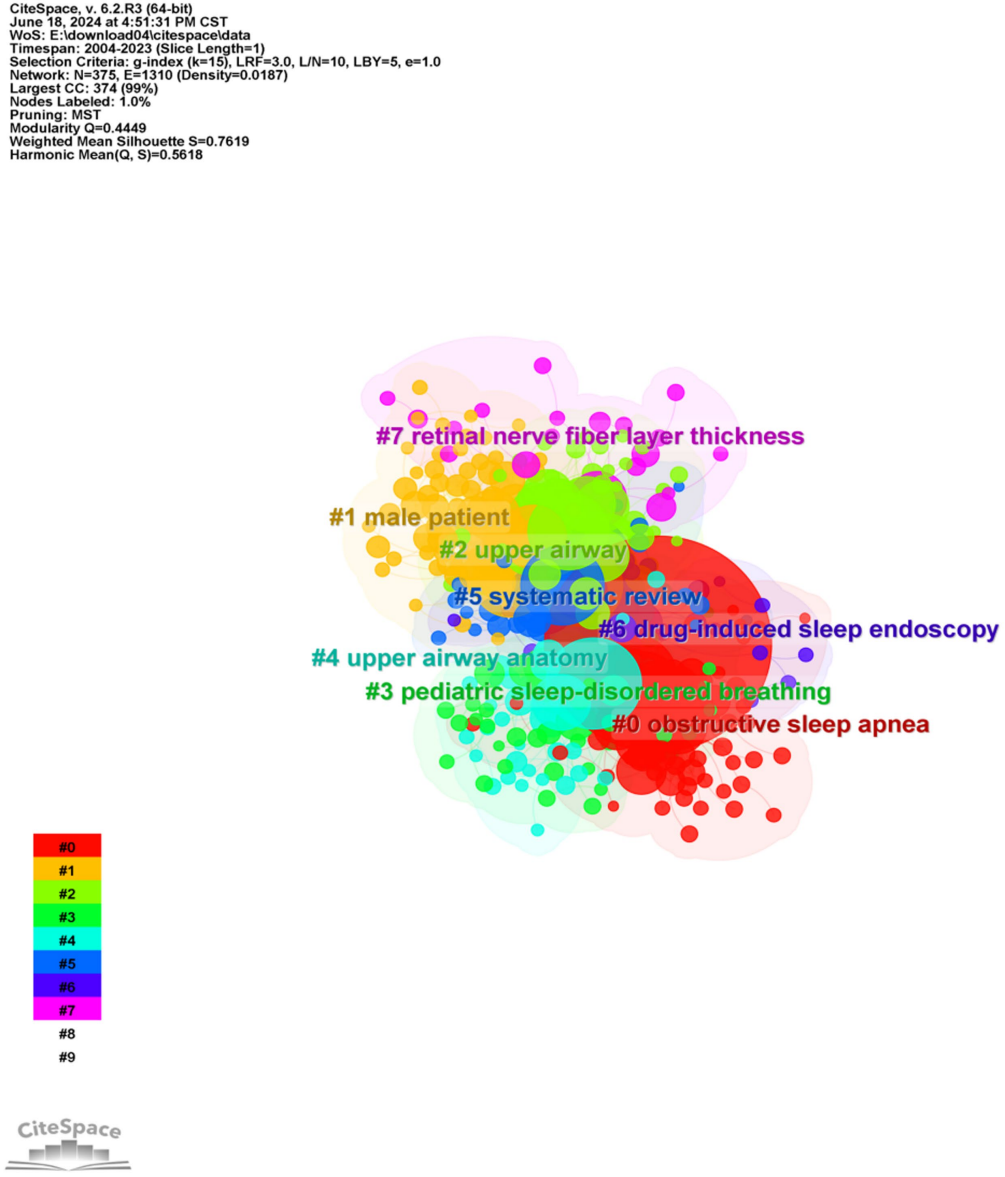
Cluster map of keywords related to OSA and radiological imaging. Created using CiteSpace.

**Figure 9:**
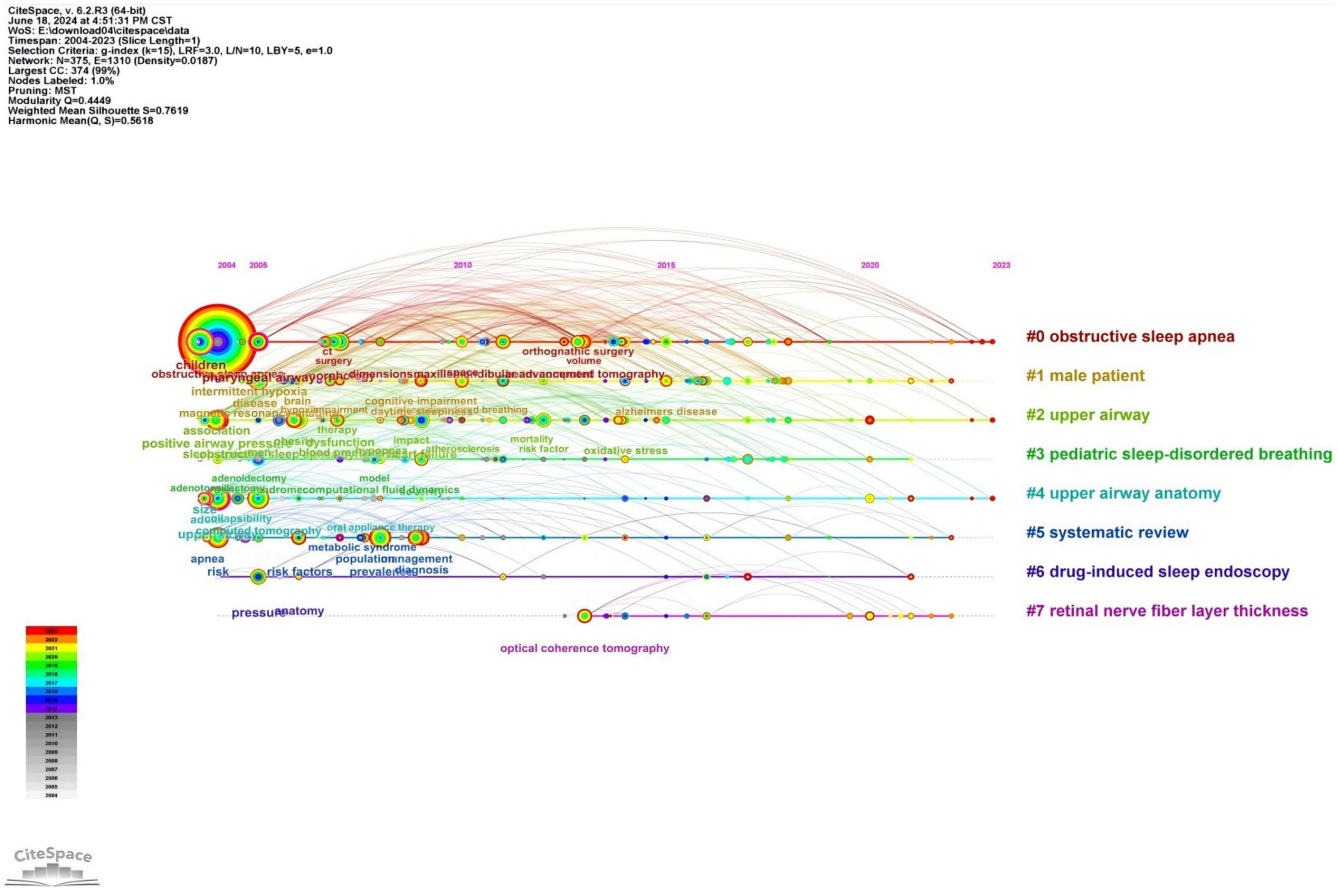
Timeline chart of keywords related to OSA and radiological imaging. Created using CiteSpace.

**Figure 10:**
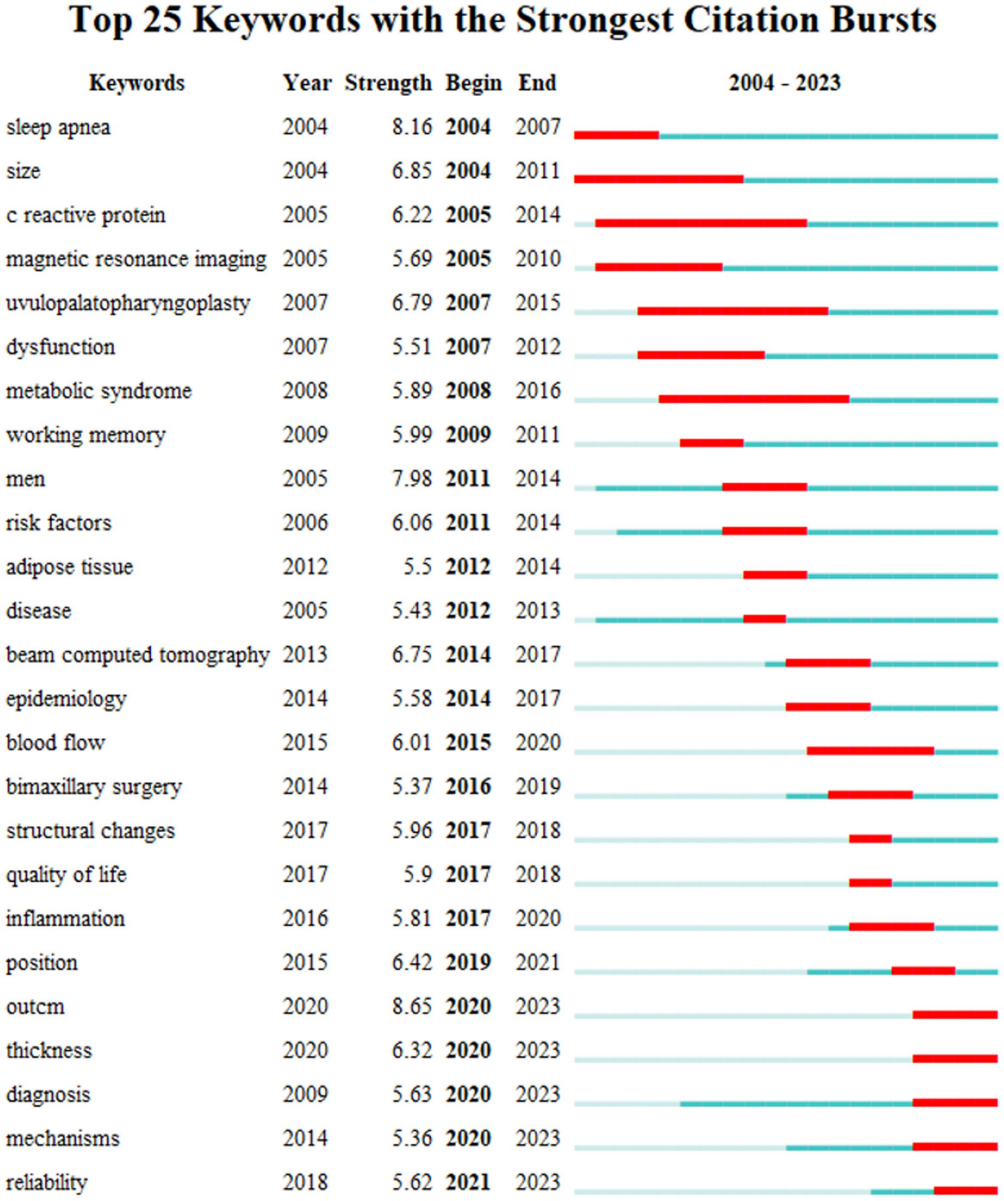
Top 25 keywords with the strongest citation bursts.

To understand the research frontiers of obstructive sleep apnea syndrome in the field of radiological imaging since 2003, CiteSpace was used to cluster the keywords related to OSA and radiological imaging. Table 4, Figures 8, 9 show eight clusters. Generally, when the silhouette score is greater than 0.5, the clustering effect is considered reasonable. Cluster #0 is labeled “obstructive sleep apnea syndrome,” followed by Cluster #1 “male patient,” Cluster #2 “upper airway,” Cluster #3 “pediatric sleep-disordered breathing,” Cluster #4 “upper airway anatomy,” Cluster #5 “systematic review,” Cluster #6 “drug-induced sleep endoscopy,” and Cluster #7 “retinal nerve fiber layer thickness,” representing the research frontiers since 2003. Burst keywords encapsulate a sudden surge in research content over a specific period, potentially signaling future research trends. Figure 10 presents the top 25 items with the highest burst strength in this research topic. The red lines in the chart denote the duration of the keyword bursts. From this chart, we can discern that the keyword themes have progressively transitioned from “sleep apnea,” “size,” and “C-reactive protein” to more recent themes such as “structural changes,” “inflammation,” “diagnosis,” and “mechanisms.” This suggests that the correlation between obstructive sleep apnea syndrome and aspects such as maxillofacial changes, imaging diagnosis, and pathological mechanisms is the central focus of both current and anticipated future research in this field.

**Table 4:** Keyword cluster analysis.

Cluster	Size	Silhouette	Mean year	Label (LLR)	Other keywords
0	69	0.77	2013	Obstructive sleep apnea	Children; morphology; pharyngeal airway; surgery; beam computed tomography; volume; orthographic surgery; dimensions; space
1	67	0.723	2011	Male patient	Magnetic resonance imaging; disease; intermittent hypoxia; sleep-disordered breathing; Alzheimer’s disease; impairment; daytime sleepiness; cognitive impairment; hypoxia; stroke
2	63	0.757	2011	Upper airway	Sleep apnea; positive airway pressure; association; obstructive sleep apnea syndrome; obesity; hypertension; heart failure; cardiovascular disease; dysfunction; blood pressure
3	46	0.707	2011	Pediatric sleep-disordered breathing	Severity; endoscopy; apnea syndrome; computational fluid dynamics; adenotonsillectomy; model; uvulopalatopharyngoplasty; adenoidectomy; quality of life; flow
4	42	0.726	2011	Upper airway anatomy	Upper airway; computed tomography; adults; size; collapsibility; outcome; resistance; fat; hypertrophy; oral appliance therapy
5	37	0.815	2010	Systematic review	Prevalence; risk; diagnosis; risk factors; apnea; management; population; metabolic syndrome; sleep disordered breathing; disorders
6	20	0.773	2011	Drug-induced sleep endoscopy	Pressure; anatomy; efficacy; update; hypopnea syndrome; mandibular advancement device; motion; experience; Chiari malformation; 3 dimensional assessment
7	19	0.862	2016	Retinal nerve fiber layer thickness	Optical coherence tomography; thickness; retinal nerve fiber layer; normal tension glaucoma; fiber layer thickness; choroidal thickness; idiopathic intracranial hypertension; OSAS; cerebrospinal fluid; optical coherence tomography angiography

## 4 Discussion

### 4.1 General information discussion

The objective of this study was to collate all pertinent WoSCC data to identify research areas of significance and emerging trends within the field. The number of publications has been increasing at a steady rate on an annual basis. The United States was the most prolific nation in terms of published articles, with a total of 704, followed by China and Turkey. The United States occupies a dominant position in this field due to its robust economic strength, favorable policies, and robust scientific support. Meanwhile, although China is a developing country, it has the highest number of patients with obstructive sleep apnea syndrome (OSAHS) in the world, at 176 million. In the context of specific research fields, bibliometrics can be employed to assess the extent of collaboration among authors, institutions, and countries (20). The concept of centrality is used to quantify the degree of collaboration. The countries with the highest centrality are the United States, China, Canada, Sweden, and Australia, indicating that these countries are actively engaged in collaborative research with a range of other nations.

The most closely collaborating institutions in obstructive sleep apnea (OSA) imaging research include the University of California system, Harvard University, the University System of Ohio, Capital Medical University, and Seoul National University (SNU). While these institutions demonstrate formal partnerships, author centrality metrics remain at 0, revealing fundamental collaboration deficits. Notably, influential contributors such as Kumar R., Harper RM., Schwab RJ., Macey PM., and Woo MA. exhibit minimal co-authorship patterns, suggesting systemic barriers to knowledge exchange rather than individual reluctance. Two major obstacles hinder global research synergies:

(1) Diagnostic disparities: Previous studies have found inconsistent OSA diagnostic criteria between Asian and European populations, including differences in diagnostic equipment and threshold variations.
(2) Methodological heterogeneity: The 2017 American Academy of Sleep Medicine guidelines indicate that portable monitoring devices may underestimate the Apnea-Hypopnea Index (AHI), while laboratory polysomnography (PSG) is more accurate. Racial differences in upper airway morphology further complicate research comparisons—a 2020 MRI study showed that Chinese populations have smaller total airway dimensions and all measurement parameters of the retropalatal (RP) airway and retroglottal (RG) airway compared to Icelandic populations at the same BMI levels (21). Despite recognition of environmental and genetic regulatory factors (such as the different effects of Icelandic cold adaptation versus Southeast Asian humid and hot environments on airway reactivity), existing international alliances still lack standardized protocols for multi-ethnic studies. The field urgently needs to establish FAIR (Findable, Accessible, Interoperable, Reusable) data principles and equitable funding allocation mechanisms.

The fostering of collaboration among authors necessitates the existence of a supportive environment, characterized by cooperation across related research fields and the implementation of government policies conducive to such endeavors. We contend that close collaboration among countries, institutions, and authors will contribute to significant progress in this field.

### 4.2 Current status and trends of OSA in the field of imaging

Performing keyword analysis to summarize the themes and research directions of Obstructive Sleep Apnea Hypopnea Syndrome (OSAHS) within the field of medical imaging is crucial. Our research findings suggest that research topics related to the pathophysiological mechanisms and comorbidities of OSAHS have become increasingly concentrated and interconnected in recent years. This suggests that interest in this research field is becoming more convergent, and the relationships between different topics are becoming closer. Our keyword analysis underscores that the imaging assessment of upper airway structures in relation to Obstructive Sleep Apnea Hypopnea Syndrome (OSAHS) has emerged as a focal point of extensive discourse in the field. Polysomnography is the gold standard for diagnosing OSAHS and grading its severity8. However, due to limitations such as venue and personnel, only a few hospitals can conduct it, resulting in a high prevalence but low diagnosis rate of OSAHS. Imaging plays an important role in evaluating the repetitive collapse of the upper airway and studying the anatomical factors related to the pathogenesis of OSAHS. Different imaging modalities have different focuses on the diagnosis and treatment of OSA patients.

#### 4.2.1 Imaging evaluation of adult obstructive sleep apnea syndrome

Cephalometric X-ray, being the simplest imaging examination, is currently the most widely used tool in sleep medicine for evaluating the upper airway. Radiologists use X-rays to measure and analyze various landmarks of the dentition and craniofacial structures, thus understanding the structural characteristics and interrelationships of the soft and hard tissues of the dentition and craniofacial region (22). However, in current clinical practice, the primary task of cephalometric analysis is often performed by experienced radiologists. The influence of personal clinical experience, reading conditions, and measurement habits may lead to variability in research results, and lacking standardization. The development of modern deep learning technology has effectively addressed this issue. Originating from the study of artificial neural networks (ANN), deep learning aims to form more abstract high-level category attributes or features by combining low-level features to discover data distribution patterns (23). Using this technology to assist in image reading can significantly reduce the workload of clinicians, improve the accuracy of landmark detection and measurement, and establish a standardized analysis model that aligns with clinical practice through large-sample mechanical deep learning.

At present, the application trends of deep learning technology in the diagnosis and treatment of Obstructive Sleep Apnea (OSA) can be distilled into three primary facets: determining the timing of OSAHS treatment, automatic recognition and measurement of cephalometric landmarks, and making medical decisions related to OSAHS. This aims to familiarize radiologists with deep learning and provide clinical doctors with references for the rational selection of CPAP pressure titration during treatment.

Using CT technology, three-dimensional images of the craniofacial and upper airway anatomy can be obtained. Compared to cephalometric X-rays, CT scans significantly improve soft tissue contrast, allowing precise measurement of the cross-sectional area of various regions, three-dimensional reconstruction, and airway volume assessment. In recent years, CT technology has been widely used to evaluate pharyngeal narrowing in OSA patients while awake. Additionally, for narrow areas that are difficult to observe in two-dimensional images (24), radiologists can further analyze using CBCT, which is currently the most effective, rapid, and low-radiation-dose method. CBCT not only has high spatial resolution but also provides clear contrast between soft tissues and cavities in the images. Compared to CT, CBCT has a lower radiation dose, with the radiation dose of CBCT being only one-fifth of that of CT under the same quality requirements. Moreover, CT can be combined with portable polysomnography (PSG) to assess the upper airway condition in OSA patients. Chousangsuntorn et al. (25)found that using CT (combined with portable PSG) to scan the upper airway of OSA patients during apnea episodes, compared to when awake, can yield better anatomically and pathologically relevant images of OSA. With the increasing popularity of artificial intelligence, Kim et al. (26). Developed a new deep learning model, including a multimodal deep learning model and an airway prominence pre-processing algorithm obtained from CT images used for other purposes, which can provide significantly accurate results for the diagnosis of obstructive sleep apnea (OSA).

Additionally, CT also holds value in the research of OSA comorbidities. Lee et al. (27)found through OCT imaging of the optic disc that OSAHS patients are at risk of developing glaucoma early on. Daniel et al. (28)concluded through lumbar spine CT scans of OSA patients that, after controlling for age, gender, and cardiovascular disease, obstructive sleep apnea syndrome is associated with changes in bone density, further speculating on the complex impact of obstructive sleep apnea syndrome on bone density and the potential clinical significance of vertebral compression or femoral neck fractures. Lu et al. (29) discovered through coronary CTA that obstructive sleep apnea is independently associated with the presence and burden of coronary artery plaques, with a higher incidence of coronary artery plaques in patients with moderate to severe OSA. Furthermore, studies have found a significant correlation between OSA and the incidence of subclinical atherosclerosis (30), indicating an increased risk of coronary events in OSA patients.

In conclusion, there is increasing evidence that OSA increases the risk of cardiovascular events (31, 32). Therefore, in future research, using coronary CTA to detect undiagnosed OSA patients will be a future development trend.

MRI, with its advantages of multi-parameter, multi-sequence, multi-orientation, high sensitivity, and no radiation, plays an important role in evaluating the upper airway soft tissue structure of OSA and studying the pathogenesis and comorbidities of OSAHS. Volumetric MRI is a powerful tool for identifying and quantifying anatomical risk factors for obstructive sleep apnea. Richard et al. (19) found through volumetric MRI that during obstructive sleep apnea events, the probability of airway closure in the retropalatal region is higher than in the retroglossal region. This may be due to the anatomical narrowing of the airway caused by the increased volume of the pharyngeal lateral walls in this region. Furthermore, after adjusting for confounding factors (race, gender, age, craniofacial size, and visceral fat in the neck), it was found that the risk of sleep apnea increases with the volume of the tongue, pharyngeal lateral walls, and total soft tissue.

Using MRI elastography and chemical shift imaging techniques (such as Dixon or m-Dixon), radiologists can delve deeper into the evaluation of extrinsic tongue muscle function and fat deposition in patients suffering from Obstructive Sleep Apnea Syndrome (OSAHS) (33, 34). It was found that one of the pathogenic factors of OSA is obesity-related tongue fat deposition, which impairs the function of the genioglossus muscle. As muscle tension decreases, the probability of oropharyngeal obstruction during sleep increases. Moreover, deep (as opposed to subcutaneous) fat deposition in the genioglossus muscle is the main reason why the prevalence of OSA is twice as high in men as in women. Furthermore, the escalating incidence of Obstructive Sleep Apnea observed with advancing age can be attributed to factors such as the accumulation of adipose tissue and a decline in muscular functionality.

In addition to the advantages of conventional MRI imaging, dynamic MRI also has good temporal resolution, allowing the observation of dynamic changes in airway morphology. Dynamic MRI can evaluate the airway at different stages of breathing and can also study physiological parameters during induced or natural sleep (35–37). It is the only imaging technique that can simultaneously show apnea or hypopnea with PSG. It is also an intuitive method for evaluating OSAHS patients and determining the specific location of airway obstruction (38). Additionally, dynamic MRI is used to assess changes in the upper airway of OSA patients treated with mandibular advancement devices to evaluate treatment efficacy (39).

With the recent accelerated advancements in functional Magnetic Resonance Imaging (fMRI), the focus of future research in this domain may shift toward unraveling the pathophysiological mechanisms underpinning the cognitive dysfunction associated with Obstructive Sleep Apnea (OSA). In corresponding neuroimaging investigations, both conventional and functional brain imaging methodologies have been employed to explore the neural consequences of untreated Obstructive Sleep Apnea (OSA). Investigations employing Magnetic Resonance Imaging (MRI) in middle-aged and older demographics have identified moderate to severe Obstructive Sleep Apnea (OSA) as an independent risk determinant for white matter disease (40). Further meta-analysis also shows that 70% of patients with transient ischemic attacks or ischemic or hemorrhagic strokes had previously undiagnosed OSA (41). Recent applications of Diffusion Tensor Imaging (DTI), an advanced MRI technique, have revealed diminished integrity of white matter fibers across various brain regions in patients with Obstructive Sleep Apnea (OSA) (42). Remarkably, a full reversal of these white matter anomalies was observed following a 12-month treatment regimen with Continuous Positive Airway Pressure (CPAP) (42). These investigations propose that the cognitive impairments observed in Obstructive Sleep Apnea (OSA) could be attributed to injuries in the brain regions responsible for executing these tasks. Although traditional neuroimaging biomarkers, such as decreased gray matter volume, altered white matter integrity, and abnormal functional connectivity, have provided important evidence for OSA-related cognitive impairments, these single-modality imaging techniques often only capture static, isolated changes in brain structure or function. Recent developments in neuroimaging primarily focus on multimodal fusion imaging technology: ingeniously combining functional magnetic resonance imaging (fMRI) with real-time electroencephalography (EEG) monitoring techniques to achieve dynamic, real-time, multidimensional mapping of neural circuits disrupted by OSA. This fusion technology not only simultaneously records blood oxygen level-dependent signals and electrophysiological activities of the brain but also allows real-time observation of immediate effects on neural networks during apnea events in OSA patients, thereby precisely identifying critical components of functional abnormalities. While technologies such as fMRI may provide new perspectives for the study of the neural mechanisms of sleep disorders, the information obtained from a single research model is still limited and cannot fully reflect the flow of information in brain networks and neural circuits. At present, countries worldwide are advancing research in this field, aiming to precisely construct and deeply analyze different dimensions of brain networks in sleep disorders through integrating novel and traditional brain function research methods, such as polysomnography and multimodal imaging technologies. Combined with cohort follow-up studies, these efforts aim to deepen our understanding of the physiological changes in brain function related to sleep disorders and associated diseases, promote innovation in sleep disorder intervention techniques, and safeguard the improvement of diagnosis and treatment levels for sleep disorders.

#### 4.2.2 Imaging evaluation of pediatric obstructive sleep apnea syndrome

The primary cause of Obstructive Sleep Apnea (OSA) in children is adenotonsillar hypertrophy (43). Therefore, the development of pediatric OSA imaging mainly focuses on the morphology of the upper airway in children and changes in the upper airway before and after adenotonsillectomy in children (44, 45), serving as the basis for radiological assessment.

Cephalometry is a common technique for upper airway imaging. The lateral skull radiograph offers advantages such as low radiation dose, ease of operation and acquisition, and low cost, making it highly suitable for assessing the craniofacial structure and upper airway size in younger children (3). Lateral skull radiographs help screen children at high risk for OSA (46), providing a relatively reliable basis for early intervention and treatment in children at high risk for OSA. Despite many advantages, cephalometry has limitations. First, X-rays are taken while the patient is awake and standing, so it cannot fully assess the collapsibility of the patient’s upper airway during sleep. Second, the low resolution of X-ray planar films affects the clarity of researchers observing whether there are abnormalities in the patient’s upper airway soft tissues. In addition, the two-dimensional nature of X-ray planar films cannot accurately display the three-dimensional structure of the upper airway, which may lead to inconsistencies in diagnosis by radiologists.

Three-dimensional reconstruction with CT can quickly and accurately assess the pharyngeal breathing space in children, but the high radiation and high cost of CT three-dimensional reconstruction significantly reduce its usage rate (47). Even though Cone Beam CT (CBCT) has less radiation than CT, its clinical use is still very limited (48).

Previous studies on pediatric OSA have demonstrated the clinical efficacy of MRI in assessing abnormal upper airways in children and OSA. Zeng et al. (49) found in their evaluation study using MRI that the ratio of adenoid area to nasopharyngeal area in children was positively correlated with the obstructive apnea-hypopnea index (*r* = 0.922, *p* < 0.001) and negatively correlated with the lowest SpO_2_ value (*r* = −0.858, *p* < 0.001). Furthermore, Nandalike et al. (50) found in their study using upper airway MRI imaging that there was significant residual adenoid tissue after adenotonsillectomy in pediatric OSA patients, and the volumes of the soft palate and tongue increased. This could partially explain the residual OSA in obese pediatric OSA patients after adenotonsillectomy. In the pediatric population, MRI also has limitations. Unlike CT, MRI is noisy, takes a long time to acquire, and is more expensive than other imaging methods. Unlike most adults, children under 6 years old usually need sedation or anesthesia to tolerate the entire MRI study. Moreover, because of the sedation, the patient’s airway under this state cannot represent the airway situation under normal sleep (51).

The results of this bibliometric analysis have several implications for future research. First, strengthening cooperation between researchers and institutions is needed to promote innovation and comprehensive research. Second, attention to advanced imaging technologies should be maintained and expanded, including emerging technologies that can provide deeper insights. Finally, interdisciplinary research combining clinical, imaging, and molecular approaches may offer holistic solutions to address OSA and its potential sequelae.

### 4.3 Advantages and limitations of research

This investigation utilizes bibliometric techniques to scrutinize articles published over an approximately two-decade span (2004–2023), to discern global patterns and emergent research focal points in the domain of medical imaging for Obstructive Sleep Apnea Syndrome (OSA). The intention is to furnish insights that can guide future scientific inquiries. Nevertheless, it is crucial to acknowledge certain constraints of this study. First, the research is contingent on the Web of Science (WoS) core database, excluding other databases, which may inadvertently lead to the exclusion of pertinent literature, thereby introducing a degree of selection bias. Second, the study is confined to English language publications, potentially exacerbating language and publication bias. Lastly, while the study’s principal emphasis is on adult sleep apnea syndrome, offering a broad perspective of the field by evaluating countries, journals, institutions, authors, and keywords, it might neglect crucial, nuanced aspects of the developmental trends of pediatric obstructive sleep apnea syndrome in the realm of medical imaging. Given these limitations, future studies should aim to widen the study population, encompass a broader array of databases, incorporate non-English publications, and delve into more granular aspects of the imaging field for obstructive sleep apnea syndrome. This approach will facilitate a more comprehensive and precise comprehension of this field.

## 5 Conclusion

This investigation offers an exhaustive review and evaluation of literature from 2003 to 2022 about Obstructive Sleep Apnea (OSA) and medical imaging, thereby unearthing prevailing research trajectories and focal points in this domain. Our findings suggest an escalating trend in the annual publication count, indicative of burgeoning research activity in this field, particularly in nations such as the United States, China, and Turkey, which are leading the charge in OSA and imaging research. Furthermore, our study underscores the necessity for bolstering collaboration among prominent institutions and authors.

Our keyword analysis sheds light on the primary research themes in this domain, inclusive of the correlation between OSA and the upper airway, as well as their association with radiology. Moreover, our examination has unearthed emerging research trends, such as the nexus between OSA, maxillofacial modifications, imaging diagnosis, and pathological mechanisms, which are anticipated to be the focal points of imminent research.

Our study also provides an overview of the current landscape and trends of OSA in the imaging field. We discovered that research topics associated with the pathophysiological mechanisms and comorbidities of Obstructive Sleep Apnea Hypopnea Syndrome (OSAHS) have become increasingly focused and interconnected in recent years. This implies a converging interest in this research domain and an intensifying interrelation among various topics. Our keyword analysis emphasizes the significance of the upper airway structure in the imaging evaluation of OSAHS. In light of the rapid advancements in functional Magnetic Resonance Imaging (fMRI), future research may pivot toward uncovering the pathophysiological mechanisms underlying cognitive dysfunction linked to OSA. Nevertheless, the existing body of scientific literature remains inadequate for a thorough understanding of the pathogenesis of cognitive impairment associated with OSA. Further imaging studies are required to elucidate the relationship between OSA and cognitive deficits.

This bibliometric analysis yields several implications for forthcoming research. Firstly, efforts should be made to enhance collaboration between researchers and institutions to foster innovation and holistic research. Secondly, continued and expanded focus should be placed on advanced imaging technologies, inclusive of nascent technologies that can offer profound insights. Lastly, interdisciplinary research integrating clinical, imaging and molecular approaches may furnish comprehensive solutions to tackle OSA and its potential consequences.

## Data Availability

The original contributions presented in the study are included in the article/supplementary material, further inquiries can be directed to the corresponding author.
